# Hippocampal metabolic differences implicate distinctions between physical and psychological stress in four rat models of depression

**DOI:** 10.1038/s41398-017-0018-1

**Published:** 2018-01-10

**Authors:** Lanxiang Liu, Xinyu Zhou, Yuqing Zhang, Juncai Pu, Lining Yang, Shuai Yuan, Libo Zhao, Chanjun Zhou, Hanping Zhang, Peng Xie

**Affiliations:** 1 Department of Neurology, The First Affiliated Hospital of Chongqing Medical University, Chongqing, China; 2 Institute of Neuroscience and The Collaborative Innovation Center for Brain Science, Chongqing Medical University, Chongqing, China; 3 Department of Psychiatry, The First Affiliated Hospital of Chongqing Medical University, Chongqing, China; 4 Department of Neurology, Yongchuan Hospital of Chongqing Medical University, Chongqing, China

## Abstract

Major depressive disorder (MDD) is a heterogeneous and multi-factorial disorder, and the underlying molecular mechanisms remain largely unknown. However, many studies have indicated that the molecular mechanisms underlying depression in response to different stress may differ. After screening, 28–30 rats were included in each model of depression (chronic unpredictable mild stress (CUMS); learned helplessness (LH); chronic restraint stress (CRS); or social defeat (SD)). Non-targeted gas chromatography-mass spectrometry was used to profile the metabolic changes in the hippocampus. As a result, all four models exhibited significant depression-like behavior. A total of 30, 24, 19, and 25 differential metabolites were identified in the CUMS, LH, CRS, and SD models, respectively. Interestingly, the hierarchical clustering results revealed two patterns of metabolic changes that are characteristic of the response to cluster 1 (CUMS, LH) and cluster 2 (CRS, SD) stress, which represent physical and psychological stress, respectively. Bioinformatic analysis suggested that physical stress was mainly associated with lipid metabolism and glutamate metabolism, whereas psychological stress was related to cell signaling, cellular proliferation, and neurodevelopment, suggesting the molecular changes induced by physical and psychological stress were different. Nine shared metabolites were opposite in the directions of change between physical and psychological models, and these metabolites were associated with cellular proliferation and neurodevelopment functions, indicating the response to physical and psychological stress was different in the activation and deactivation of the final common pathway to depression. Our results provide a further understanding of the heterogeneity in the molecular mechanisms of MDD that could facilitate the development of personalized medicine for this disorder.

## Introduction

Pharmacotherapy and manual-driven psychotherapy are both frequently used treatments for major depressive disorder (MDD), either as monotherapies or in combination^1^. However, treatment response varies considerably between individuals: four successive treatment steps in the STAR*D trial resulted in a cumulative remission rate of only 67%^2^, and most antidepressants do not seem to offer a clear advantage for young patients^3^. Among the psychotherapies, interpersonal therapy and cognitive-behavioral therapy should be considered as the initial choice for MDD treatment^4,5^. In summary, no single treatment is likely to be effective for MDD as a whole, and only a subset of patients will respond to any given treatment.

Scientists have suggested that heterogeneity in treatment response is the direct result of etiological heterogeneity in MDD^6^. Stress plays an important role in the pathogenesis of depression^7^. However, stressors differ in their behavioral and physiological outcomes^8^. Dayas et al.^9^ proposed that the brain categorizes at least two main categories of stressor, “physical” and “psychological”, which elicit distinctive response in the brain. Kavushansky et al.^10^ proved that the pattern of hormonal responses and the expression of plasticity-related genes in the hippocampus differ in response to physical stress and psychosocial stress. Changes in hippocampal concentrations of extracellular zinc, a signaling factor in synaptic neurotransmission, differ between physical and psychological stress^11^. Accordingly, different stressors can cause heterogeneous and even diametrically opposed stress responses^12,13^. On account of that the hippocampus is a main brain region involved in the pathogenesis of MDD^14^. Therefore, we hypothesized that the pathogenetic processes involved in MDD in the hippocampus may differ between different stress.

We investigated this hypothesis by exploring hippocampal metabolic changes in four animal models of depression induced by different stress. Rats were, respectively, exposed to chronic unpredictable mild stress (CUMS)^15^, inescapable foot-shock stress (learned helplessness, LH)^10,16^, repeated restraint stress (chronic restraint stress, CRS)^17,18^, and resident-intruder stress (social defeat, SD)^10,19^ to mimic different types of stress in humans.

The aim of the present study was to investigate the metabolic changes in the hippocampus in the four stress models of depression using a non-targeted gas chromatography-mass spectrometry (GC-MS) approach. We were specifically interested in whether the molecular mechanisms in the etiology of depression induced by different types of stress differ from each other. Furthermore, we also compared the metabolic phenotypes between models to explore the final common pathway to depression.

## Materials and methods

### Animals

**Fig. 1:**
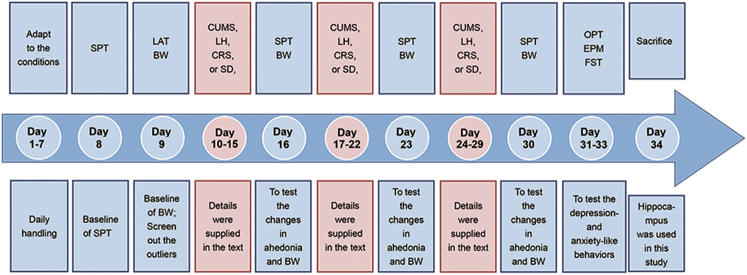
Experimental regime and timetable for the four stress paradigms CUMS chronic unpredictable mild stress, LH learned helplessness, CRS chronic restraint stress, SD social defeat, SPT sucrose preference test, BW body weight, LAT locomotor activity test, OFT open field test, EPM elevated plus-maze, FST forced swimming test

One hundred forty male Sprague-Dawley rats with initial weights of 200–300 g were single-housed and maintained in standard conditions with a reverse 12-h light/12-h dark cycle (lights on at 1900 hours; lights off at 0700 hours) at a constant temperature (22 ± 1 °C) and relative humidity (55 ± 5%). Food and water were available ad libitum throughout the experiments except where noted. The experiments began after 1 week of habituation to the housing conditions. Each individual model included 28–30 rats after screening. The screening process was performed as previously described^20,21^. The remaining rats were then randomly allocated to the stress or control group. The schedule for the experimental procedure is provided in Fig. 1. The treatment of animals and the procedures were in accordance with the National Institutes of Health guidelines^22^ and approved by the Ethics Committee of Chongqing Medical University.

### CUMS model

A typical depression phenotype caused by chronic mild stress was modeled in rats using a CUMS paradigm. Rats in the stress group were exposed to varying stressors performed randomly on a daily basis for 3 weeks. The stressors in the CUMS regime were based on previously published protocols^15,23,24^, including restraint for 4 h, water/food deprivation for 24 h, cold stress at 4 °C for 1 h, tail pinch for 1 min, swim stress in water at 18 °C for 5 min, light on for 12 h, light off for 3 h, stroboscope for 12 h, reversal of the light/dark cycle for 24 h, cage tilting for 24 h, crowding for 24 h, wet bedding for 24 h. The CUMS protocol of the study is provided in Supplementary Table S1. The control rats were handled daily.

### LH model

In the LH paradigm, a stressed rat was placed in one side of a shuttle box (Academy of Medical Sciences, Shandong, China) and exposed to 60 inescapable foot-shocks (0.85 mA intensity, 15 s average duration, 15 s average inter-shock interval). Rats in the control group were placed in the chambers for an equivalent time with no electric foot-shocks. Learned helpless behavior—latency to escape and escape failures—were evaluated by active escape testing consisting of 30 trials of escapable foot-shocks (0.8 mA intensity, 10-s maximum duration, 30-s average inter-trial interval) after 5 min of habituation. This LH protocol has been reported elsewhere^20^.

### CRS model

The CRS paradigm was used to mimic a subtype of depression caused by restraint stress, which is a critical risk factor in the etiology of depression. Rats in the stress group were repeatedly placed in plastic restrainers (550 ml cubage water bottle, Nongfu Spring Company Limited, HangZhou, China) for 6 h (from 0900 to 1500 hours) at the same time every day. This stress paradigm continued for 21 days. During the restraint stress period, rats in both the stress group and the control group were deprived of food and water. The details were reported in our previous study^21^.

### SD model

The SD paradigm, modified from the resident-intruder model^25^, was used to model stress-related depression in rats. SD was initiated when an “intruder” (Sprague-Dawley rat) was introduced into the home cage of the “resident” (aggressive male Long-Evans rat, weighing 380–450 g). The interaction continued until the intruder received a serious defeat, characterized by surrendering or acquiring a supine position for approximately 5 s, with a maximal interaction time of 5 min. Subsequently, the intruder was transferred to a wire mesh protection cage (10 × 10 × 15 cm) within the resident’s cage that allowed intense visual, auditory, and olfactory contact with the resident but prevented direct physical contact. This protective procedure lasted for 55 min. The control rats were exposed to the empty home cage of an aggressive Long-Evans rat for 60 min. The SD protocol has been reported elsewhere^26^.

### Behavioral tests

The locomotor activity test (LAT), sucrose preference test (SPT), forced swimming test (FST), open field test (OFT), and elevated plus-maze (EPM) were conducted as previously described^20,21,27^. Briefly, the LAT was used to identify rats’ activity levels prior to stress. Locomotor activity was indexed as the distance traveled (centimeters) in an open-field apparatus during a 5-min test. The SPT was conducted weekly. Sucrose preference, calculated as sucrose intake/total fluid intake (water + sucrose) during the 1-h test, was used as a measure of anhedonia in rats. Body weight was measured weekly immediately after the SPT. Total immobility time in the FST was recorded for 5 min as an index of depression-like behavior. Spatial exploration behavior in rodents was tested by the OFT, locomotor activity, central activity, and rearing frequency measured during the 5-min session. The number of entries into and time (s) spent in the open and closed arms of an EPM were assessed as a measure of anxiety-like behavior over 5 min.

### GC-MS analysis of rat hippocampus samples

Hippocampus samples were obtained from stressed rats and corresponding controls after being anaesthetized by an intraperitoneal injection of 10% chloral hydrate (100 g/0.4 ml). For each depression model, an independent pool of case and control hippocampi was created. Metabolic profiling of the processed hippocampi was achieved using an Agilent 7890A/5975C GC/MSD System (Agilent Technologies Inc., USA). Details of the pretreatment of the hippocampus samples and GC-MS analysis were provided in our previous studies^20,21^.

### Statistical analyses

The results of behavioral tests were expressed as the mean ± SEM. Comparisons of behavioral characteristics were performed by SPSS 21.0 (IBM, New York, USA) using two-sample Student’s *t*-tests or non-parametric Mann−Whitney *U*-tests, as appropriate. *P*-values < 0.05 were considered significant. Metabolomics analysis was performed using several software programs. Principal component analysis (PCA) and orthogonal partial least-squares discriminant analysis (OPLS-DA) were performed using SIMCA-P + 11.5 software (Umetrics, Umeå, Sweden). Metabolites with variable importance in the projection (VIP) values >1 in the OPLS-DA model were preliminarily considered significantly different, then validated at a univariate level using Student’s *t*-test followed by multiple testing using the Benjamini–Hochberg procedure with the critical false discovery rate (FDR) set to 0.05. Those with an FDR < 0.05 were selected as significantly differential metabolites. Heat maps and unsupervised hierarchical clustering analysis of hippocampal differential metabolites were constructed using Matlab (Mathworks, Natick, MA, USA). Differential metabolites (with PubChem CIDs) along with their fold changes were subsequently uploaded to the Ingenuity database for pathways and dominant networks analysis using the Ingenuity Pathway Analysis software (IPA, http://www.ingenuity.com).

## Results

### Quality of the animal models

The screening results are shown in Supplementary Fig. S1. The models included a total of 114 rats, in the following groups: CUMS/Control = 20/8, LH/Control = 20/8, CRS/Control = 20/10 and SD/Control = 20/8. No significant differences in locomotor activity or sucrose preference were found between the stress and control groups in each model. After stress exposure, the stressed rats were divided into susceptible and resilient subgroups based on whether their sucrose preference had decreased or not. In the present study, only susceptible rats were used for further analysis, resulting in 68 rats (CUMS/Control = 9/8, LH/Control = 10/8, CRS/Control = 8/8, and SD/Control = 9/8) with no significant differences in baseline body weight, sucrose preference, or locomotor activity between the experimental and control groups in each model.

**Fig. 2:**
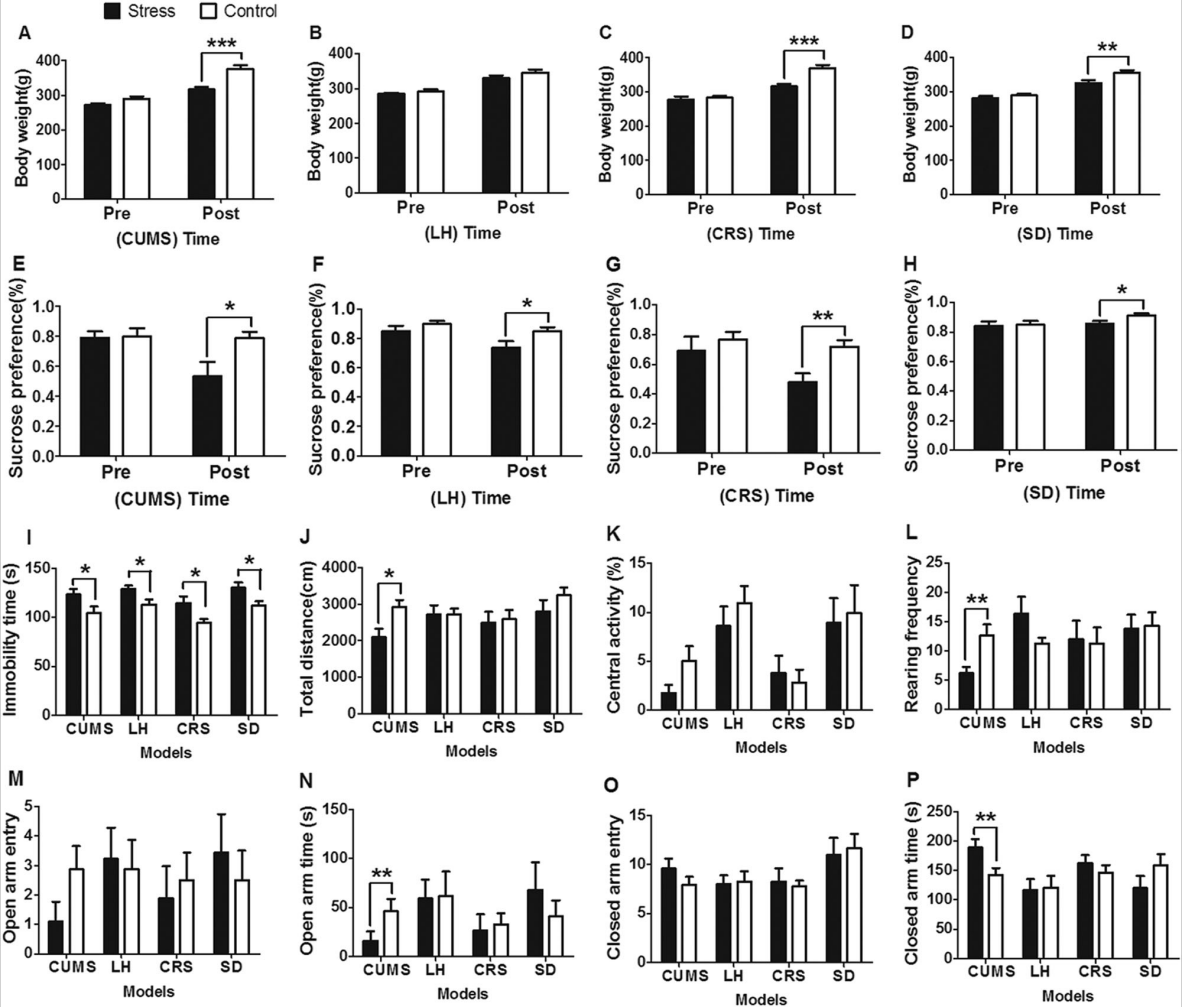
Influence of different types of stress on depression- and anxiety-like behavior **a**–**d** Body weight changes in the chronic unpredictable mild stress (CUMS), learned helplessness (LH), chronic restraint stress (CRS), and social defeat (SD) models. **e**–**h** Sucrose preference changes in the CUMS, LH, CRS, and SD models. **i** Immobility time of the four models in the forced swimming test. **j**–**l** Total distance, central activity, and rearing frequency of the four models in the open field test. **m**–**p** Total number of entries into and time spent in the open and closed arms of the four models in the elevated plus-maze. *P < 0.05, **P < 0.01, ***P < 0.001

The results of behavioral tests in the LH, CRS, and SD depression models were also reported in our previous publications, respectively^20,21,26^. Briefly, body weight gain was significantly decreased in the stressed rats compared with the corresponding controls in the CUMS (*P* < 0.001; Fig. 2a), CRS (*P* < 0.001; Fig. 2c), and SD (*P* < 0.01; Fig. 2d) models, but no statistical difference was found between the two groups in the LH model (Fig. 2b). Similarly, sucrose preference was decreased in the stressed rats in each model (CUMS, *P* < 0.05; LH, *P* < 0.05; CRS, *P* < 0.01; SD, *P* < 0.05; Figs. 2e–h). In the FST, each type of stress increased the immobility time compared with controls (Fig. 2i), indicating aggravated depression-like behavior. Moreover, the CUMS rats showed a significant decrease in total distance (*P* < 0.05; Fig. 2j) and rearing frequency (*P* < 0.05; Fig. 2l) in the OFT, with no significant effect in central activity (Fig. 2k). In comparison with controls, CUMS rats spent less time in the open arms (*P* < 0.01; Fig. 2n) and more time in the closed arms (*P* < 0.01; Fig. 2p) in the EPM, suggesting anxiety-like behavior in this model. There was no difference in the total number of entries into open or closed arms (Figs. 2m, o) between the two groups in the EPM. Taken together, the results show that each type of stress induced depression-like behavior, whereas CUMS also enhanced anxiety-like behavior.

### Metabolic changes in four depression models

**Fig. 3:**
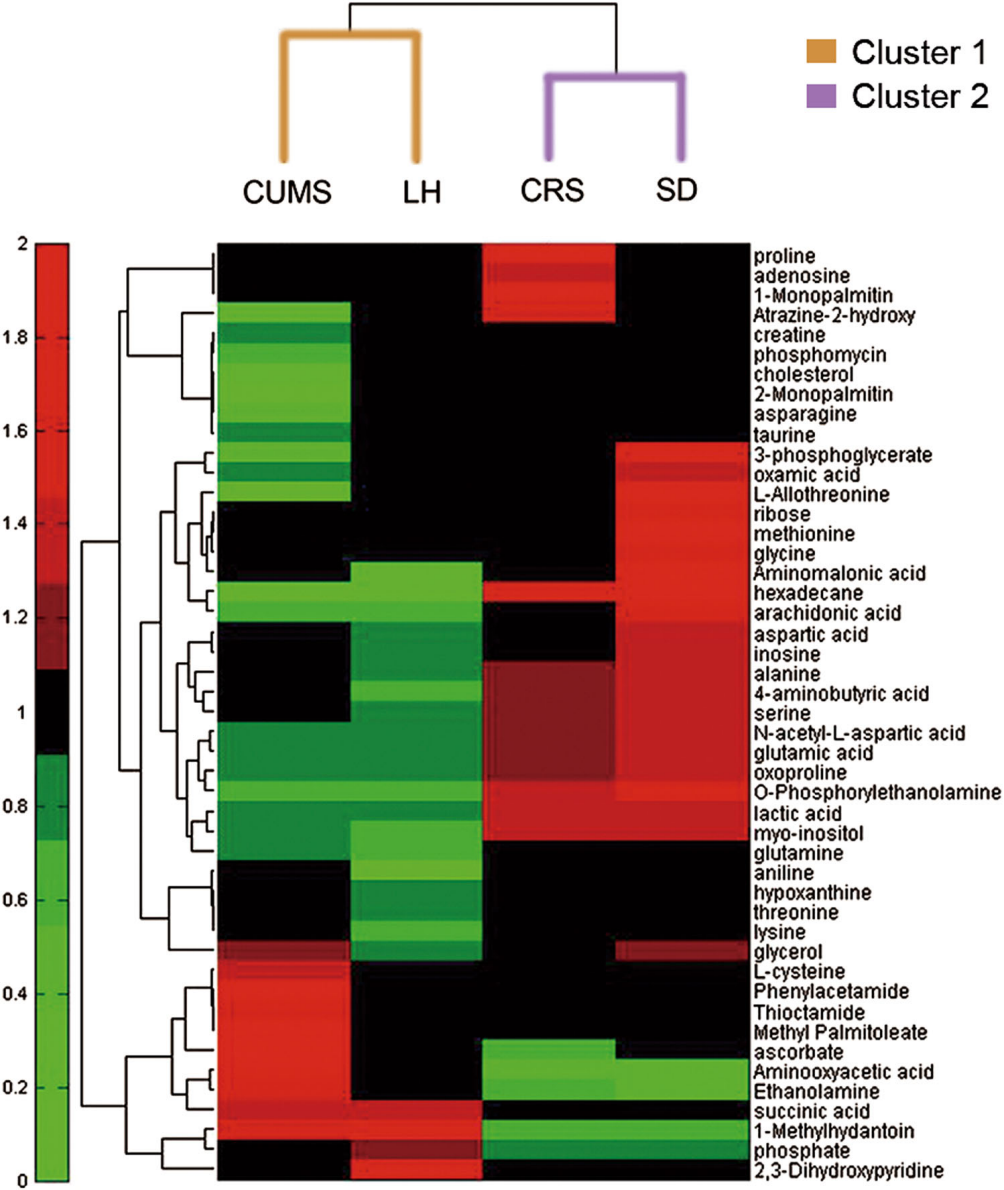
Clustering of metabolic disturbances in rat models of depression Hierarchical clustering was performed using the differentially expressed metabolites for each model based on the fold changes (stress/control)

These models feature a wide range of stress-related depression. Hippocampus samples from individual rats were analyzed at a metabolic level based on a non-targeted GC-MS approach. After data processing, quality control samples were tightly clustered on the PCA score plot (Supplementary Fig. S2), indicating that reproducibility was satisfactory. To further analyze the differences in metabolic profiles between groups, an OPLS-DA score plot was produced for individual depression models. A clear separation between two groups was observed in each model (Supplementary Fig. S3). Based on the criteria of VIP > 1 and FDR < 0.05, a total of 30, 24, 19, and 25 different metabolites were identified in the CUMS, LH, CRS, and SD models, respectively. Interestingly, these different metabolites showed similar directions of change in the CUMS and LH models, and they were opposite to those of CRS and SD. The details are shown in Supplementary Table S2. Moreover, hierarchical clustering results suggested that there are two patterns of metabolic changes that are characteristic of the response to cluster 1 (CUMS, LH) and cluster 2 (CRS, SD) stress models (Fig. 3). To further understand the underlying molecular mechanisms, functional networks analysis for each of the four models and comparative analysis between models were performed.

### Metabolic analysis of CUMS and LH depression models

**Fig. 4:**
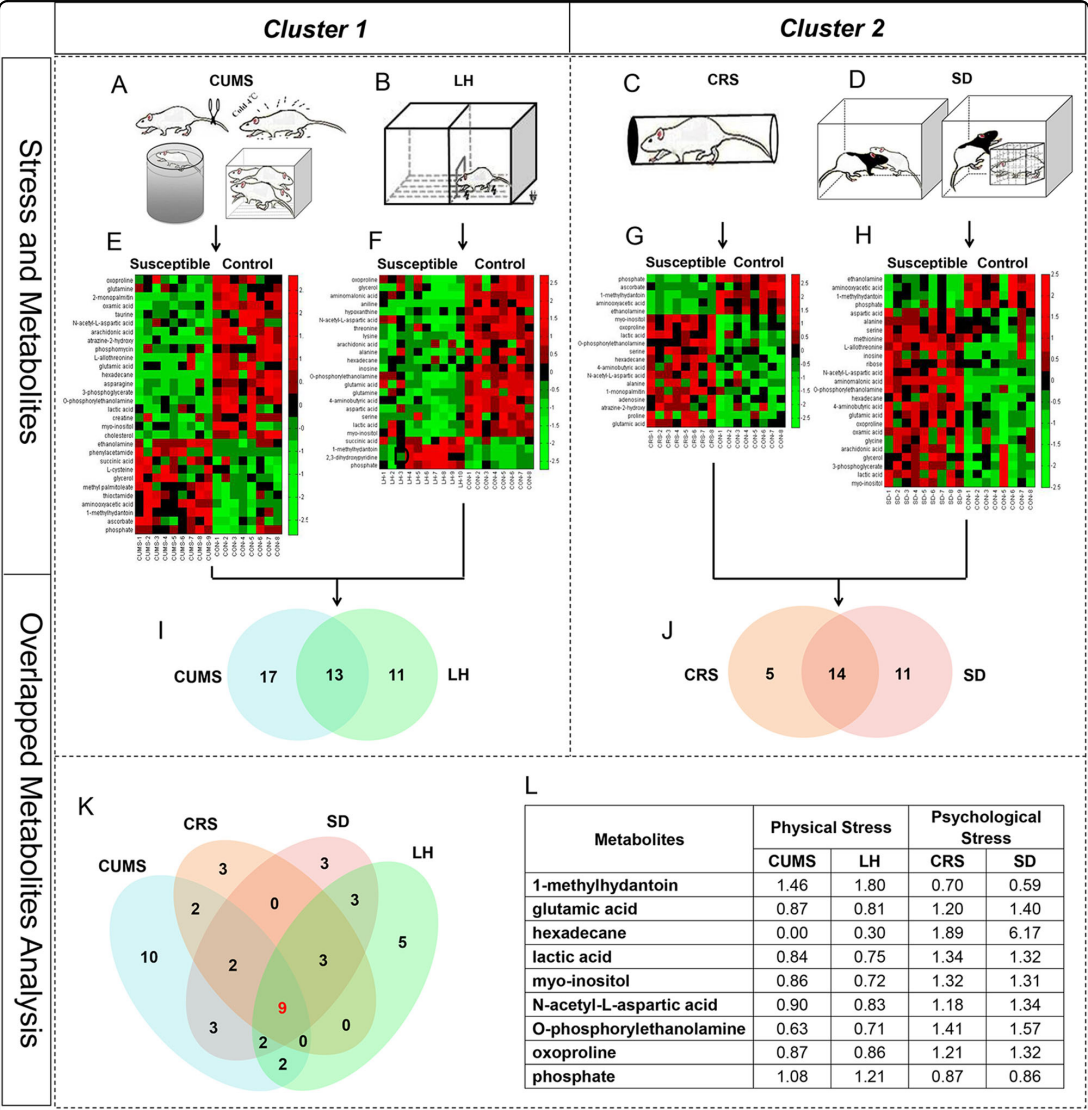
Distinct metabolic signatures in hippocampus of the four depression models **a**–**d** The stress strategies in the chronic unpredictable mild stress (CUMS), learned helplessness (LH), chronic restraint stress (CRS) and social defeat (SD) models, respectively. **e**–**h** Heat maps of differential metabolites in the CUMS, LH, CRS and SD models, respectively. **i** Venn diagram of differential metabolites that were in common or unique to the CUMS and LH rat models. **j** Venn diagram of differential metabolites that were in common or unique to the CRS and SD rat models. **k** Venn diagram of differential metabolites that were in common or unique to the four depression models. **l** The details of these nine metabolites differentially expressed across all four models

The altered metabolites in the CUMS and LH models, as well as the metabolic overlap between the two conditions, were explored (Figs. 4a, b, e, f, i). A dominant networks analysis of the significantly altered metabolites using IPA identified two networks with scores of >10 in both the CUMS and LH models. Both models showed a highly significant “lipid metabolism, molecular transport, small molecule biochemistry” network (network #2 in the CUMS model; #1 in the LH model) (Supplementary Table S3). This was the only significant network for the 13 overlapping metabolites that showed the same direction of change (except for glycerol). Glutamate metabolism was the function most associated with these overlapping metabolites (Supplementary Table S4). These common disturbances involving lipid metabolism and glutamate metabolism may reflect a common response to CUMS and LH stress in hosts and may reflect functional roles for these pathways in the development of depression.

### Metabolic analysis of CRS and SD depression models

The different metabolites in the CRS and SD models and the shared 14 metabolites showing the same direction of change were exhibited in Figs. 4c, d, g, h, j. To understand the underlying molecular mechanisms of the two paradigms, differentially expressed metabolites were analyzed using IPA. Interestingly, the “cell-to-cell signaling and interaction, cellular growth, and proliferation, nervous system development and function” network was statistically significant in the CRS model, and was also identified as the top-ranking network in the SD model (Supplementary Table S3). Consequently, these results suggest that CRS and SD stress may share common perturbed pathways associated with cell signaling, cellular proliferation, and neurodevelopment. We then repeated the analysis with the 14 overlapping metabolites. The significant network, which included nine focus metabolites from our reference dataset, was similar to network #2 in the SD model.

### Metabolic analysis of the four depression models

**Fig. 5:**
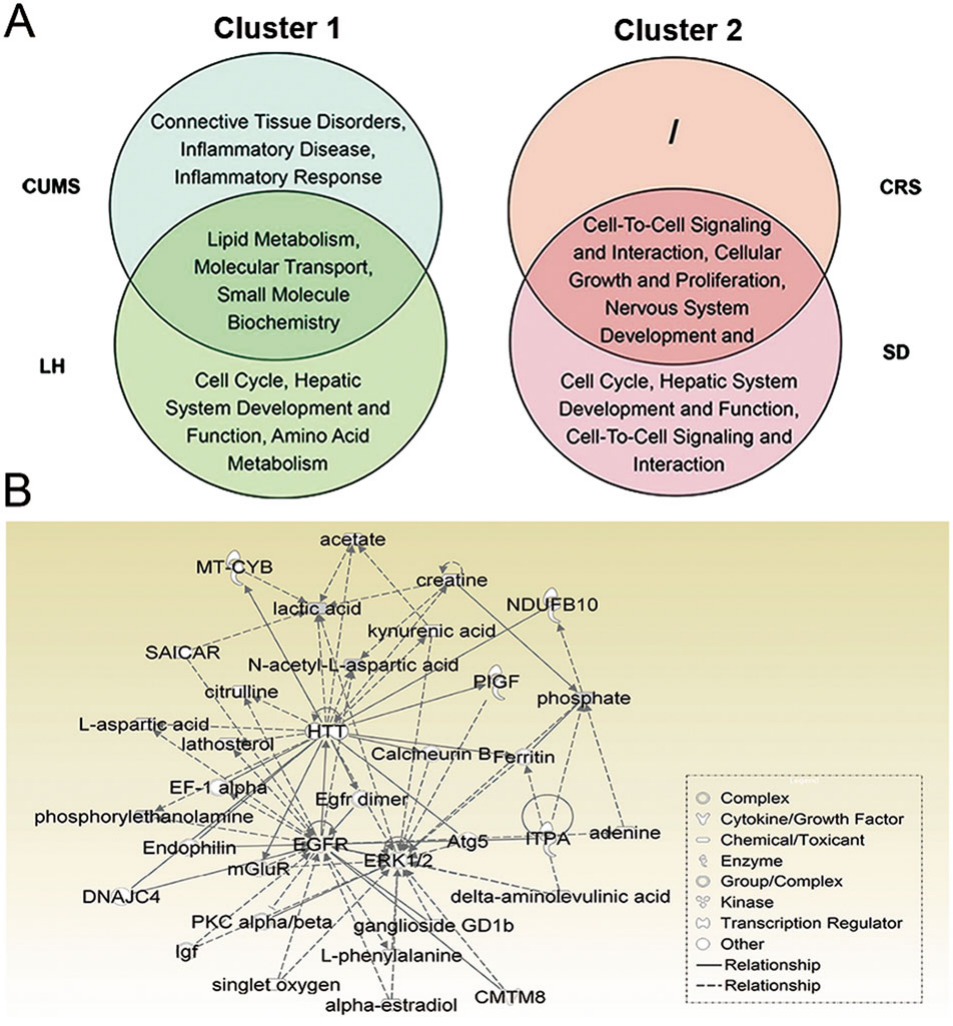
Metabolic analysis results of the two clusters depression models **a** Venn diagrams of networks that were significantly altered in cluster 1 and cluster 2 depression models. **b** The most significantly altered network based on the nine metabolites that were differentially expressed across all four models. CUMS chronic unpredictable mild stress, LH learned helplessness, CRS chronic restraint stress, SD social defeat

A compelling finding was that nine metabolites were differentially expressed across all four depression models (Figs. 4k, l). The minimal overlap in the metabolic disturbances between these models suggests that the molecular mechanisms underpinning depression caused by different types of stressors differ considerably (Fig. 5a). Interestingly, the nine overlapping metabolites changed in diametrically opposite directions between inter-clusters, indicating that the hosts’ responses to cluster 1 and cluster 2 stress involved differential activation and deactivation of a certain pathway. To provide further insight into the final common pathway to depression, we analyzed these metabolites. The perturbed pathways were mainly involved in sphingosine and sphingosine-1-phosphate metabolism, and four metabolites (lactic acid, *N*-acetyl-l-aspartic acid, phosphorylethanolamine, and phosphate) were found in the significant “cellular growth and proliferation, organismal development, nervous system development and function” network (Fig. 5b).

## Discussion

The present study compared the hippocampal metabolomics of four stress-related depression models using the non-targeted GC-MS approach. Analysis of the metabolic profiles resulted in two clusters, cluster 1 containing the CUMS and LH depression models and cluster 2 containing the CRS and SD depression models. We found that the molecular mechanisms were largely overlapping in the two models of depression in cluster 1, as well as in the two models in cluster 2, but were distinct between the two cluster types. Moreover, a small set of metabolites that showed diametrically opposite inter-cluster directions of change were differentially expressed across all four models, which may suggest that the response to cluster 1 and cluster 2 stress differs in the activation and deactivation of the final common pathway to depression.

Although these findings are interesting, they also raise the important question of why these four depression models were divided into two clusters. Considering the properties of each stressor, we inferred that the main reason may be the type of stress. The majority of the stressors (e.g., cold stress, tail pinch, and swim stress) in the CUMS regime^28–31^ and the inescapable foot-shock stress in the LH protocol^10,16,32^ were physical components, whereas repeat restraint stress in the CRS model^17,18^ and resident-intruder stress in the SD model^10,19^ mainly induce psychological stress. Taken together, cluster 1 could be considered as physical stress, and cluster 2 as psychological stress. Although both types of stress consist of a mixture of physical and psychological components, the significant differences in the composition of physical and psychological aspects of each stress may be responsible for the differences in the molecular mechanisms.

Both of the physical stress (cluster 1) models exhibited significant depression-like behavior. Analysis of each individual model and the 32% of metabolites (13/41) that overlapped between the two models indicated that lipid metabolism and glutamate metabolism may be the shared pathways in the development of physical-related depression. Disturbances in lipid metabolism and glutamatergic metabolism and neurotransmission have been widely reported in patients with MDD^33–35^. However, metabolomic analysis revealed that the functional network associated with inflammatory response was significantly disturbed only in the CUMS model. A previous study showed that the inflammatory response in the brain can affect the molecular pathways that influence the neurotransmitter systems that regulate depression- and anxiety-like behavior^36^, and elevated inflammation has also been observed in patients with current anxiety disorders^37^. Interestingly, it may be due to the variety, intensity, and duration of stressors, rats in the CUMS model exhibited several anxiety-like behavior^38,39^, and this may explain why the inflammatory response was more significant in this model.

Following psychological stress (cluster 2), the CRS and SD models exhibited a similar change tendency in significant depression-like behavior. Unsurprisingly, both models showed disturbances in the pathways associated with cell signaling, cellular proliferation, and neurodevelopment. Consistent with our finding, it has been hypothesized that impaired neurogenesis and cellular plasticity contribute to MDD pathogenesis^40,41^. Moreover, chronic psychosocial stress was found to interfere with hippocampal neurogenesis and other aspects of neuronal plasticity^42,43^. Nearly half of the metabolites (14/30) overlapped between the two models, which may reflect a common response to psychological stress and common pathways to depression. As animal models typically exhibit some features of a disease but not others^44^, these model-specific metabolites may be associated with the more distinct features of depression rather than the more common features.

The analysis of the individual model revealed heterogeneity in the biological mechanisms of depression between the physical and psychological stress models. This finding is perfectly consistent with those of previous studies suggesting that physiological responses to physical and psychological stressors differ^45,46^. Moreover, there is evidence indicating that physical and psychological stress activate distinct neural regions^47^, which could explain the differences between these two types of stress in the underlying pathogenetic response to depression in a particular region. In the hippocampus, the overlapping metabolites with opposite directions of change between inter-models may reflect the differences in the activation and deactivation of the final common pathways that respond to physical and psychological stress.

Most interestingly, of these overlapping metabolites, disturbances in glutamate, lactic acid, myo-inositol, and *N*-acetyl-l-aspartic acid have been reported in depressed patients^35,48–52^, and an antidepressive effect of 1-methylhydantoin has been shown in depressed rodents^53^. Therefore, all of these overlapping metabolites were analyzed as an independent pool to gain further understanding into the final common pathway to depression. The results revealed a significant alteration in the network that included lactic acid, *N*-acetyl-l-aspartic acid, phosphorylethanolamine, and phosphate. Lactic acid is a product of glycolysis. Mitochondrial dysfunction leading to anaerobic glycolysis has been reported in depression^54^. *N*-acetyl-l-aspartic acid levels partly reflect mitochondrial dysfunction^55^, which impairs the regulation of neurodevelopment and synaptic plasticity^56,57^—the possible pathogenesis of depression^58,59^. Moreover, phosphorylethanolamine and phosphate are mainly involved in sphingosine metabolism, which was identified as the most significantly perturbed pathway among the set of metabolites. It is well known that phosphorylethanolamine has specific effects on mitochondrial dysfunction in depression^60^.

Depression is a heterogeneous and multi-factorial disorder and diagnosed on the basis of symptoms. However, developing biomedicine-based classification, diagnose, and treatment for depression is a major feature of Precision Medicine Initiative^61^. Drysdale et al.^62^ first subdivided patients with depression into four biotypes defined by distinct patterns of dysfunctional connectivity in brain by using neuroimaging. In addition, multiple evidences suggested that, either in psychosis or other diseases, distinct biological mechanisms lead to remarkably similar clinical manifestations^63,64^. Thus, it is likely that distinct metabolic alterations in different depression models lead to the shared behavioral symptoms.

Personalized medicine promises “the right drug at the right time for the right patient”^65,66^. Meaningfully, the results of present study may have far reaching implications for personalized medicine for MDD. Our findings indicate that etiological factors (e.g., physical and psychological stressors) could be used to predict the underlying molecular mechanisms in a given depressed patient and, therefore, select the most effective treatments. Depressed individuals vary widely in their responses to specific treatments in clinical practice. This heterogeneity in treatment response may be explained by the heterogeneity in the molecular mechanisms underlying depression caused by different stressors (e.g., physical and psychological stressors). Nevertheless, further studies in clinical samples are required to confirm this hypothesis.

Some important limitations to the present study need to be mentioned. First, the relatively limited sample size in each model was a primary limitation, and may have led to false-positive findings. However, the accumulative number of four models was a large sample size, which could reflect the real changes. Second, we only used a metabolomic approach in this study, and the possibility that the single-omic data restricted the interpretation of the present results cannot be excluded. Therefore, in future studies, we will integrate proteomics and metabolomics to confirm our findings. Third, our study focused exclusively on metabolic changes in the hippocampus and did not consider other brain regions implicated in depression (e.g., the amygdala) because the hippocampus is the key brain region in the neurobiological development of depression. Fourth, categorizing these stressors into physical and psychological groups may distract from considering other aspects of these protocols. However, based on the previous studies, it may be the most appropriate and likely hypothesis. Moreover, these two types of stress were categorized on the basis of the two distinct patterns of metabolic changes, which may tend to limit the consideration of alternative possibilities. Nonetheless, it could provide an important clue to unraveling the biological differences between physical and psychological stress in depression. Last, animal models cannot capture the complex features of a disease and thus it is crucial to translate our present findings from animals to humans in future studies.

In conclusion, the results of metabolic profiling presented here demonstrate abundant metabolic changes in the context of depression. Although it remains largely unclear whether MDD consists of a heterogeneous set of features with distinct molecular mechanisms, our findings provide support for this hypothesis. Using multiple models of depression, we showed that physical and psychological stressors cause distinct molecular changes underlying depression. Moreover, we also identified a small set of differentially expressed metabolites across all four models, suggesting a final common pathway to depression. However, the responses to physical and psychological stress resulted in differential activation and deactivation of this final common pathway. Our study contributes to a better understanding of the heterogeneity in the molecular mechanisms of MDD that could facilitate the development of personalized medicine for this disease.

## Electronic supplementary material

Supplemental information
