# Supplementary material for: Hippocampal metabolic differences implicate distinctions between physical and psychological stress in four rat models of depression
Source: Transl Psychiatry. 2018 Jan 10;8:4. doi: 10.1038/s41398-017-0018-1 (PMC5802536; doi:10.1038/s41398-017-0018-1)
Supplement: Supplementary file 1 — Supplemental information [file 41398_2017_18_MOESM1_ESM.docx]

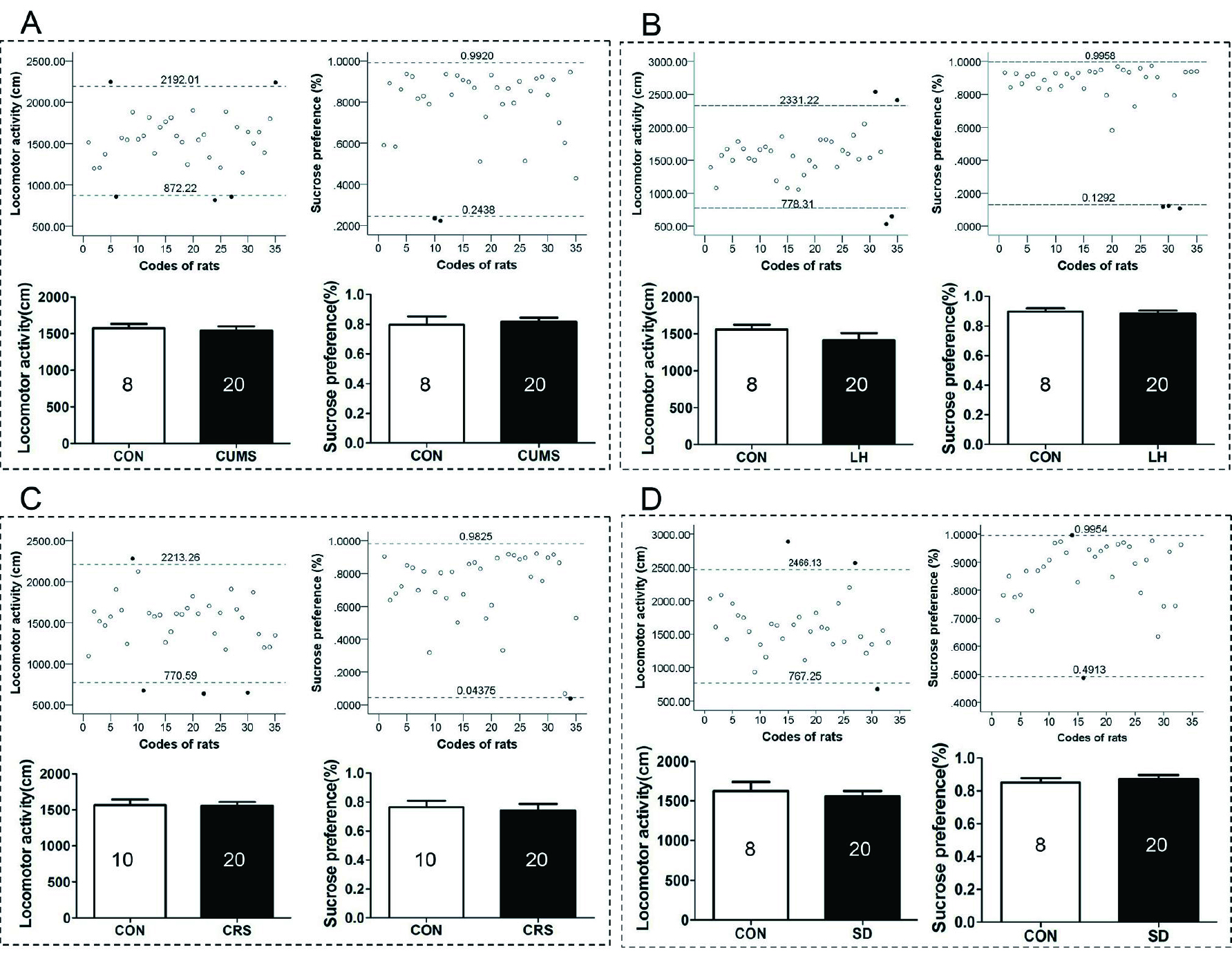


**Supplementary Figure S1**. Screening results based on the locomotor activity test (LAT) and sucrose preference test (SPT). Each model included 35 male Sprague-Dawley rats before screening. The 95% reference interval (Shapiro–Wilk test, p > 0.05) of locomotor activity in the LAT and the 95% percentile interval (Shapiro–Wilk test, p < 0.05) of sucrose preference in the SPT were used to screen for outliers. (A) Seven rats (5 on the LAT and 2 on the SPT) with spurious values (black dots) were eliminated from the chronic unpredictable mild stress (CUMS) model, leaving 20 CUMS and 8 control (CON) rats. (B) Seven rats (4 on the LAT and 3 on the SPT) with spurious values (black dots) were eliminated from the learned helplessness (LH) model, leaving 20 LH and 8 CON rats. (C) Five rats (4 on the LAT and 1 on the SPT) with spurious values (black dots) were eliminated from the chronic restraint stress (CRS) model, leaving 20 CRS and 10 CON rats. (D) Two rats with disability were excluded initially and 5 rats (3 on the LAT and 2 on the SPT) with spurious values (black dots) were eliminated from the social defeat (SD) model, leaving 20 SD and 8 CON rats. No significant statistical differences in locomotor activity or sucrose preference were found between the two groups in each model. (B) and (C) were reported in our previous studies.^1,2^

1. Zhou X, Liu L, Zhang Y, Pu J, Yang L, Zhou C, et al. Metabolomics identifies perturbations in amino acid metabolism in the prefrontal cortex of the learned helplessness rat model of depression. Neuroscience 2017; 343: 1-9.
2. Liu L, Zhou X, Zhang Y, Liu Y, Yang L, Pu J, et al. The identification of metabolic disturbances in the prefrontal cortex of the chronic restraint stress rat model ofdepression. Behav Brain Res 2016; 305:148-156.


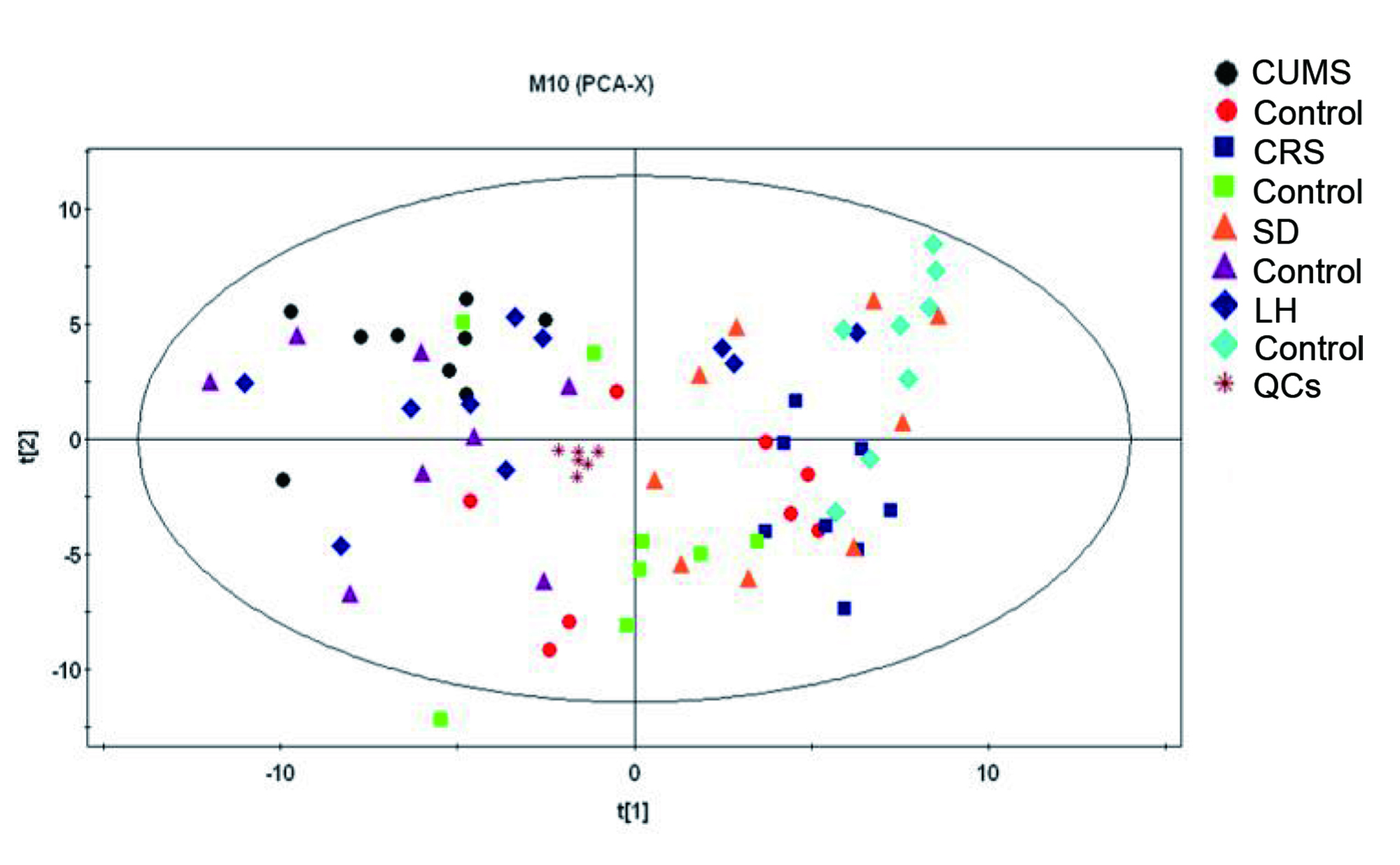


**Supplementary Figure S2**. Principal component analysis (PCA) score plot of hippocampi from the chronic unpredictable mild stress (CUMS), learned helplessness (LH), chronic restraint stress (CRS) and social defeat (SD) models of depression. Quality control samples (QCs) were clustered tightly on the PCA score plot, indicating satisfactory reproducibility.


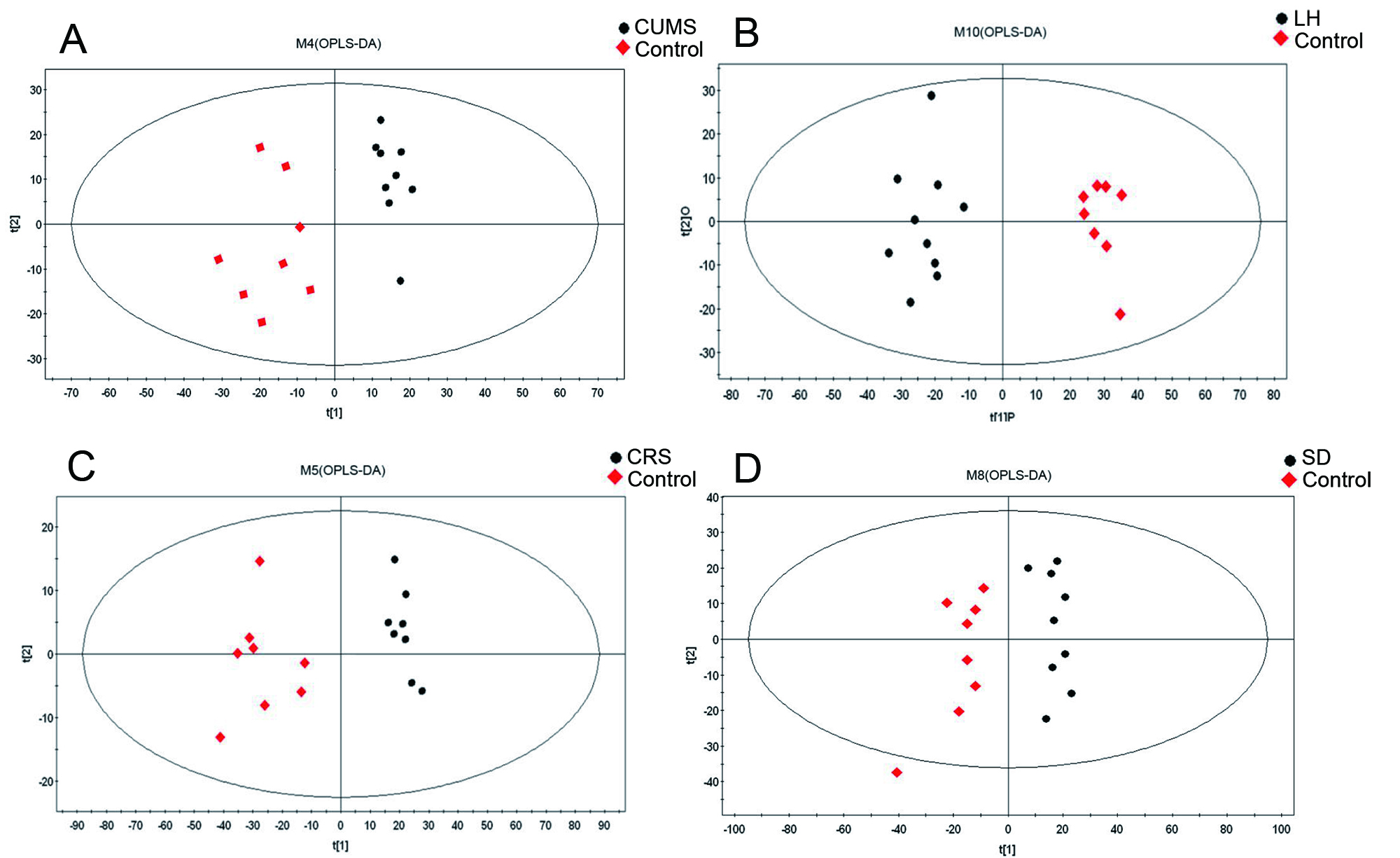


**Supplementary Figure S3**. Orthogonal partial least-squares discriminant analysis (OPLS-DA) score plots of stressed rats and corresponding controls in the individual depression model. (A) OPLS-DA score plot for chronic unpredictable mild stress (CUMS) model (R^2^X = 0.811, R^2^Y = 0.866 and Q^2^ = 0.79). (B) OPLS-DA score plot for learned helplessness (LH) model (R^2^X = 0.933, R^2^Y = 0.991, and Q^2^ = 0.880). (C) OPLS-DA score plot for chronic restraint stress (CRS) model (R^2^X = 0.814, R^2^Y = 0.857 and Q^2^ = 0.688). (D) OPLS-DA score plot for social defeat (SD) model (R^2^X = 0.896, R^2^Y = 0.890 and Q^2^ = 0.762). A clear separation between two groups was observed in each model.

**Supplementary Table S1.** Schedule for the chronic unpredictable mild stress procedure

|  | **Day1** | **Day2** | **Day3** | **Day4** | **Day5** | **Day6** | **Day7** |
| --- | --- | --- | --- | --- | --- | --- | --- |
| **Week 1** | restraint for 4h,  stroboscope for 12h | water deprivation for 24h, reversal of the light/dark cycle for 24h | cold stress at 4℃ for 1h, wet bedding for 24h | tail pinch for 1min,  food deprivation for 24h | swim stress in water at 18℃ for 5min, cage tilting for 24h | light on for 12h,  crowding for 24h | food deprivation for 24h, light off for 3h |
| **Week 2** | cold stress at 4℃ for 1h,wet bedding for 24h | light on for 12h,  crowding for 24h | swim stress in water at 18℃ for 5min, cage tilting for 24h | restraint for 4h,  stroboscope for 12h | water deprivation for 24h, reversal of the light/dark cycle for 24h | tail pinch for 1min,  stroboscope for 12h | food deprivation for 24h, light off for 3h |
| **Week 3** | tail pinch for 1min,  stroboscope for 12h | cold stress at 4℃ for 1h, wet bedding for 24h | light on for 12h,  crowding for 24h | swim stress in water at 18℃ for 5min, cage tilting for 24h | water deprivation for 24h, reversal of the light/dark cycle for 24h | restraint for 4h,  stroboscope for 12h | food deprivation for 24h, light off for 3h |

**Supplementary Table S2.** List of the differentially expressed metabolites in the hippocampi of the four depression models

| **Metabolites** | **CUMS model** | | | **LH model** | | | **CRS model** | | | **SD model** | | |
| --- | --- | --- | --- | --- | --- | --- | --- | --- | --- | --- | --- | --- |
|  | **FC** | **FDR** | **VIP** | **FC** | **FDR** | **VIP** | **FC** | **FDR** | **VIP** | **FC** | **FDR** | **VIP** |
| **Overlap across four models** | | | | | | | | | | | | |
| 1-Methylhydantoin | 1.46 | 0.0059 | 1.57 | 1.80 | 0.0019 | 1.96 | 0.70 | 0.0016 | 1.24 | 0.59 | 0.0048 | 1.41 |
| glutamate | 0.87 | 0.0141 | 1.69 | 0.81 | 0.0079 | 2.19 | 1.20 | 0.0084 | 1.53 | 1.40 | 0.0111 | 2.25 |
| hexadecane | 0.00 | 0.0002 | 1.77 | 0.30 | 0.0140 | 1.47 | 1.89 | 0.0165 | 1.75 | 6.17 | 0.0043 | 1.66 |
| lactic acid | 0.84 | 0.0440 | 2.64 | 0.75 | 0.0018 | 4.68 | 1.34 | 0.0028 | 3.29 | 1.32 | 0.0200 | 4.67 |
| myo-inositol | 0.86 | 0.0408 | 5.51 | 0.72 | 0.0025 | 5.59 | 1.32 | 0.0011 | 6.40 | 1.31 | 0.0285 | 6.56 |
| N-acetyl-L-aspartic acid | 0.90 | 0.0135 | 1.17 | 0.83 | 0.0020 | 1.17 | 1.18 | 0.0049 | 1.20 | 1.34 | 0.0122 | 1.30 |
| O-Phosphorylethanolamine | 0.63 | 0.0002 | 2.38 | 0.71 | 0.0011 | 1.06 | 1.41 | 0.0022 | 1.67 | 1.57 | 0.0046 | 1.68 |
| oxoproline | 0.87 | 0.0456 | 3.18 | 0.86 | 0.0343 | 6.78 | 1.21 | 0.0061 | 2.96 | 1.32 | 0.0111 | 4.33 |
| phosphate | 1.08 | 0.0199 | 7.65 | 1.21 | 0.0026 | 6.54 | 0.87 | 0.0006 | 8.79 | 0.86 | 0.0043 | 7.83 |
| **Overlap across three models** | | | | | | | | | | | | |
| glycerol | 1.19 | 0.0176 | 1.96 | 0.85 | 0.0093 | 1.82 | **...** | **…** | **…** | 1.21 | 0.0418 | 1.22 |
| arachidonic acid | 0.70 | 0.0040 | 1.21 | 0.71 | 0.0145 | 1.28 | **...** | **…** | **…** | 1.54 | 0.0461 | 1.22 |
| Aminooxyacetic acid | 1.72 | 0.0010 | 1.35 | **...** | **...** | **...** | 0.49 | 0.0045 | 1.02 | 0.47 | 0.0200 | 1.14 |
| Ethanolamine | 1.60 | 0.0482 | 1.13 | **...** | **...** | **...** | 0.58 | 0.0011 | 1.00 | 0.54 | 0.0177 | 1.09 |
| 4-aminobutyric acid | **...** | **…** | **…** | 0.70 | 0.0001 | 2.31 | 1.21 | 0.0061 | 1.46 | 1.42 | 0.0013 | 1.79 |
| alanine | **...** | **…** | **…** | 0.90 | 0.0301 | 1.39 | 1.11 | 0.0174 | 1.67 | 1.33 | 0.0455 | 2.46 |
| serine | **...** | **…** | **…** | 0.86 | 0.0113 | 4.78 | 1.17 | 0.0059 | 1.58 | 1.32 | 0.0480 | 2.56 |
| **Overlap across two models** | | | | | | | | | | | | |
| glutamine | 0.79 | 0.0274 | 1.98 | 0.61 | 0.0015 | 1.89 | **...** | **…** | **…** | **...** | **...** | **...** |
| succinic acid | 1.42 | 0.0290 | 1.36 | 1.32 | 0.0447 | 1.15 | **...** | **…** | **…** | **...** | **...** | **...** |
| ascorbate | 1.90 | 0.0130 | 2.14 | **...** | **...** | **...** | 0.58 | 0.0162 | 2.78 | **...** | **...** | **...** |
| Atrazine-2-hydroxy | 0.07 | 0.0003 | 1.33 | **...** | **...** | **...** | 1.51 | 0.0169 | 1.06 | **...** | **...** | **...** |
| oxamic acid | 0.77 | 0.0001 | 1.02 | **...** | **...** | **...** | **...** | **…** | **…** | 1.32 | 0.0142 | 1.00 |
| 3-phosphoglycerate | 0.39 | 0.0002 | 2.54 | **...** | **...** | **...** | **...** | **…** | **…** | 1.65 | 0.0336 | 2.08 |
| L-Allothreonine | 0.12 | 0.0013 | 1.42 | **...** | **...** | **...** | **...** | **…** | **…** | 7.70 | 0.0006 | 1.08 |
| Aminomalonic acid | **...** | **…** | **…** | 0.27 | 0.0001 | 1.04 | **...** | **…** | **…** | / | 0.0000 | 1.42 |
| aspartate | **...** | **…** | **…** | 0.77 | 0.0002 | 3.45 | **...** | **…** | **…** | 1.31 | 0.0296 | 2.05 |
| inosine | **...** | **…** | **…** | 0.75 | 0.0025 | 1.61 | **...** | **…** | **…** | 1.32 | 0.0350 | 1.21 |
| **Model specific** | | | | | | | | | | | | |
| L-cysteine | 1.35 | 0.0126 | 1.53 | **...** | **...** | **...** | **...** | **…** | **…** | **...** | **...** | **...** |
| Methyl Palmitoleate | 1.93 | 0.0043 | 1.00 | **...** | **...** | **...** | **...** | **…** | **…** | **...** | **...** | **...** |
| Phenylacetamide | / | 0.0067 | 1.00 | **...** | **...** | **...** | **...** | **…** | **…** | **...** | **...** | **...** |
| phosphomycin | 0.68 | 0.0264 | 1.34 | **...** | **...** | **...** | **...** | **…** | **…** | **...** | **...** | **...** |
| taurine | 0.81 | 0.0075 | 1.11 | **...** | **...** | **...** | **...** | **…** | **…** | **...** | **...** | **...** |
| Thioctamide | 1.45 | 0.0062 | 1.38 | **...** | **...** | **...** | **...** | **…** | **…** | **...** | **...** | **...** |
| asparagine | 0.49 | 0.0009 | 2.07 | **...** | **...** | **...** | **...** | **…** | **…** | **...** | **...** | **...** |
| cholesterol | 0.22 | 0.0003 | 3.35 | **...** | **...** | **...** | **...** | **…** | **…** | **...** | **...** | **...** |
| creatine | 0.88 | 0.0076 | 3.94 | **...** | **...** | **...** | **...** | **…** | **…** | **...** | **...** | **...** |
| 2-Monopalmitin | 0.04 | 0.0002 | 1.00 | **...** | **...** | **...** | **...** | **…** | **…** | **...** | **...** | **...** |
| aniline | **...** | **…** | **…** | 0.04 | 0.0001 | 1.09 | **...** | **…** | **…** | **...** | **...** | **...** |
| lysine | **...** | **…** | **…** | 0.64 | 0.0000 | 1.2 | **...** | **…** | **…** | **...** | **...** | **...** |
| threonine | **...** | **…** | **…** | 0.88 | 0.0053 | 1.19 | **...** | **…** | **…** | **...** | **...** | **...** |
| 2,3-Dihydroxypyridine | **...** | **…** | **…** | 2.62 | 0.0058 | 1.31 | **...** | **…** | **…** | **...** | **...** | **...** |
| hypoxanthine | **...** | **…** | **…** | 0.73 | 0.0018 | 1.11 | **...** | **…** | **…** | **...** | **...** | **...** |
| proline | **...** | **…** | **…** | **...** | **...** | **...** | 4.55 | 0.0362 | 1.03 | **...** | **...** | **...** |
| 1-Monopalmitin | **...** | **…** | **…** | **...** | **...** | **...** | 2.24 | 0.0161 | 1.04 | **...** | **...** | **...** |
| adenosine | **...** | **…** | **…** | **...** | **...** | **...** | 1.43 | 0.0109 | 1.00 | **...** | **...** | **...** |
| ribose | **...** | **…** | **…** | **...** | **...** | **...** | **...** | **…** | **…** | 1.57 | 0.0159 | 1.03 |
| methionine | **...** | **…** | **…** | **...** | **...** | **...** | **...** | **…** | **…** | 1.97 | 0.0134 | 1.09 |
| glycine | **...** | **…** | **…** | **...** | **...** | **...** | **...** | **…** | **…** | 1.59 | 0.0480 | 1.44 |

CUMS, unpredictable chronic mild stress; LH, learned helplessness; CRS, chronic restraint stress; SD, social defeat; FC, fold change; FDR, false discovery rate; VIP, variable importance in the projection.

**Supplementary Table S3.** Network analysis in the rat depression models.

| **ID** | **Score** | **Focus Molecules** | **Top Functions** | **Molecules in Network** |
| --- | --- | --- | --- | --- |
| **CUMS model** | | | | |
| **1** | **32** | **12** | **Connective Tissue Disorders, Inflammatory Disease,**  **Inflammatory Response** | ADCY, Akt, Alp, AMPK, arachidonic acid, ascorbic acid, cholesterol, creatine, creatine kinase, CYP, cytochrome C, ERK, ERK1/2, glycerol, HDL, Insulin, L-cysteine, L-glutamine, lactic acid, Ldh(complex), LDL, Mapk, N-acetyl-L-aspartic acid, NADPH oxidase, NMDA Receptor, P38 MAPK, phosphate, PI3K (complex), Pka, Pro-inflammatory Cytokine, Sod, succinate dehydrogenase, succinic acid, taurine, Vegf |
| **2** | **14** | **6** | **Lipid Metabolism,**  **Molecular Transport,**  **Small Molecule Biochemistry** | 2-methoxyestradiol, 2-palmitoylglycerol, 3-phosphoglycerate, AV3, CCKAR, CCKBR, CCLl, Cdc42, ceramide, D-galactosamine, Dgk, Dynamin, E2f, EGF, EGFR, ethanolamine, G-protein beta, Ggt, GPER1, IFNGR1, Jnk, JUN/JUNB/JUND, L-asparagine, MGAT3, NEU1, NFkB (complex), Pdgfr, phosphorylethanolamine, PIK3CA, PLA2G1B, oxoproline, SAAl, SERPINA3, stearic acid, TLR5 |
| **LH model** | | | | |
| **1** | **25** | **10** | **Lipid Metabolism,**  **Molecular Transport,**  **Small Molecule Biochemistry** | 17-hydroxyprogesterone, 5-hydroxyeicosatetraenoic acid, Akt, Alp, AQP7, arachidonic acid, ceramide-1-phosphate, CYP2E1  D-mannose, dihydroxyacetone, ERK1/2, GABA, GIP, glycerol, Grik, Insulin, Jnk, L-aspartic acid, L-glutamine, L-lysine, L-serine, L-threonine, Iactic acid, malonic acid, Mapk, MAS1, P38 MAPK, PDGF (family), phosphate, PI3K (complex), Pif, Pla2g2a, Proinsulin, pyridoxal phosphate, T-type Calcium Channel |
| **2** | **22** | **9** | **Cell Cycle, Hepatic System Development and Function,**  **Amino Acid Metabolism** | AIFl, C/ebp, C1QA, C4A/C4B, CCND1, CNR2, creatine, creatinine, Dgk, dimethylamine, EGFR, EMP3, FABP7, FTL, glycine, GPC1, HPRT1, HTT, hypoxanthine, inosine, L-alanine, L-aspartic acid, L-serine, L-threonine, Mt1, N-acetyl-L-aspartic acid, nitrite, PC, phosphorylethanolamine, SDHB, SERPING1, succinate dehydrogenase, succinic acid, transglutaminase, TSPO |
| **CRS model** | | | | |
| **1** | **35** | **12** | **Cell-To-Cell Signaling and Interaction, Cellular Growth and Proliferation, Nervous System Development and Function** | 3'-adenylic acid, adenosine, Akt, arginase, ascorbic acid, CAV1, CCND1, CDKN1A, Cyb5r3, dehydroascorbic acid, EGFR, ERK1/2, ethanolamine, GABA, glutamine, Grik, guanosine, Insulin, Irs3, Jnk, L-alanine, L-proline, L-serine, lactic acid, malonic acid, Mapk, N(omega)-hydroxyarginine, N-acetyl-L-aspartic acid, NFkB(complex), P38 MAPK, phosphate, phosphorylethanolamine, PIK3CA, Pro-inflammatory Cytokine, oxoproline |
| **SD model** | | | | |
| **1** | **26** | **10** | **Cell-To-Cell Signaling and Interaction, Cellular Growth and Proliferation, Nervous System Development and Function** | 3'-5'-ADP, 5-hydroxyeicosatetraenoic acid, Akt, Alp, arachidonic acid, ceramide-l-phosphate, CPO, D-mannose, dihydroxyacetone, ERK1/2, GABA, glycerol, glycine, Grik, Insulin, Jnk, L-aspartic acid, L-methionine, L-serine, lactic acid, Ldh (complex), malonic acid, Mapk, MAS1, N-arachidonylglycine, NMDA Receptor, phosphate, phosphocreatine, Pif, Pla2g2a, Proinsulin, pyridoxal phosphate, ribose, T-type Calcium Channel |
| **2** | **22** | **9** | **Cell Cycle, Hepatic System Development and Function, Cell-To-Cell Signaling and Interaction** | 3-phosphoglycerate, ADAR, AHNAK, AIFl, ALCAM, AREG, BGN, C4A/C4B, CCND1, CDK7, CHKA, creatinine, dinoprost, EGFR, EREG, ethanolamine, F2RL1, glutamine, glycine, GPC1, GRP, inosine, KL, L-alanine, L-aspartic acid, L-serine, Mt1, N-acetyl-L-aspartic acid, ODC1, PHGDH, phosphorylethanolamine, platelet activating factor, TSH, uric acid, Wnt |
| **From the CUMS with LH overlap** | | | | |
| **1** | **27** | **9** | **Lipid Metabolism, Molecular Transport, Small Molecule Biochemistry** | 17-hydroxyprogesterone, 5-hydroxyeicosatetraenoic acid, acetate, arachidonic acid, ceramide-1-phosphate, creatine, D-mannose, Dgk, dimethylamine, EGFR, ERBB2, ERK1/2, FABP, ganglioside GM3, glycerol, HTT, Insulin, KISS1R, L-glutamine, lactic acid, Mapk, Mlcp, Muscarinic cholinergic receptor, N-acetyl-L-aspartic acid, NTSR2, phosphate, phosphorylethanolamine, Pif, PIK3CA, Pla2g2a, psychosine, oxoproline, SAICAR, sPla2, succinic acid |
| **From the CRS with SD overlap** | | | | |
| **1** | **27** | **9** | **Cell Cycle, Hepatic System Development and Function, Carbohydrate Metabolism** | acetate, AMP, Caspase 3/7, CAV1, CCNDl, CDKNlA, CHKA, creatine, Cyb5r3, DRD3, ERK1/2, ethanolamine, FBP2, Ferritin, GABA, glycine, Grik, HCRT, hexokinase, HTT, L-alanine, L-serine, lactic acid, malonic acid, MT-CYB, N-acetyl-L-aspartic acnd, NDUFB10, niacinamide, nicotinic acetylcholine receptor, PFK, phosphate, phosphorylethanolamine, PIK3CA, oxoproline, T-type Calcium Channel |
| **From the 4 rat model overlap** | | | | |
| **1** | **11** | **4** | **Cellular Growth and Proliferation, Organismal Development, Nervous System Development and Function** | acetate, adenine, alpha-estradiol, Atg5, Calcineurin B, citrulline, CMTM8, creatine, delta-aminolevulinic acid, DNAJC4, EF-1 alpha, EGFR, Egfr dimer, Endophilin, ERK1/2, Ferritin, ganglioside GD1b, HTT, Igf, ITPA, kynurenic acid, L-aspartic acid, L-phenylalanine, lactic acid, lathosterol, mGluR, MT-CYB, N-acetyl-L-aspartic acid, NDUFB10, phosphate, phosphorylethanolamine, PIGF, PKC alpha/beta, SAICAR, singlet oxygen |

The direction of change is represented by color: red represents an increase, green represents a decrease, and orange represents the opposite directions of change between models. CUMS, chronic unpredictable mild stress; LH, learned helplessness; CRS, chronic restraint stress; SD, social defeat.

**Supplementary Table S4.** Top five significantly altered canonical pathways in the rat depression models

| **Canonical pathways** | **p-value** | **overlap** | **metabolites** |
| --- | --- | --- | --- |
| **CUMS model** | | | |
| γ-glutamyl Cycle | 1.32E-06 | 10.3% (3/29) | L-cysteine, phosphate, oxoproline |
| Anandamide Degradation | 5.43E-06 | 40.0% (2/5) | arachidonic acid, ethanolamine |
| Glutamine Biosynthesis I | 1.14E-05 | 28.6% (2/7) | L-glutamine, phosphate |
| Taurine Biosynthesis | 1.52E-05 | 25.0% (2/8) | L-cysteine, taurine |
| Asparagine Biosynthesis I | 1.52E-05 | 25.0% (2/8) | L-asparagine, L-glutamine |
| **LH model** | | | |
| tRNA Charging | 3.61E-11 | 7.3% (6/82) | L-alanine, L-aspartic acid, L-glutamine, L-lysine, L-serine, L-threonine |
| Adenine and Adenosine Salvage III | 2.96E-07 | 17.6% (3/17) | hypoxanthine, inosine, phosphate |
| Uridine-5'-phosphate Biosynthesis | 4.21E-07 | 15.8% (3/19) | L-aspartic acid, L-glutamine, phosphate |
| Purine Ribonucleosides Degradation to Ribose-1-phosphate | 4.95E-07 | 15.0% (3/20) | hypoxanthine, inosine, phosphate |
| Adenosine Nucleotides Degradation II | 1.42E-06 | 10.7% (3/28) | hypoxanthine, inosine, phosphate |
| **CRS model** | | | |
| Cysteine Biosynthesis III (mammalia) | 5.26E-07 | 9.7% (3/31) | adenosine, L-serine, phosphate |
| Phosphatidylethanolamine Biosynthesis III | 2.64E-06 | 40.0% (2/5) | ethanolamine, L-serine |
| Superpathway of Methionine Degradation | 5.06E-06 | 4.6% (3/65) | adenosine, L-serine, phosphate |
| Adenine and Adenosine Salvage I | 9.48E-06 | 22.2% ( 2/9) | adenosine, phosphate |
| tRNA Charging | 1.02 E-05 | 3.7% (3/82) | L-alanine, L-proline, L-serine |
| **SD model** | | | |
| tRNA Charging | 2.48E-09 | 6.1% (5/82) | glycine, L-alanine, L-aspartic acid, L-methionine, L-serine |
| Folate Transformations I | 3.54E-09 | 14.3% (4/28) | glycine, L-methionine, L-serine, phosphate |
| Folate Polyglutamylation | 2.44E-07 | 16.7% (3/18) | glycine, L-serine, phosphate |
| Superpathway of Serine and Glycine Biosynthesis I | 2.89E-07 | 15.8% (3/19) | glycine, L-serine, phosphate |
| Glycine Betaine Degradation | 5.27E-07 | 13.0% (3/23) | glycine, L-methionine, L-serine |
| **From the CUMS with LH overlap** | | | |
| Glutamine Biosynthesis I | 3.02E-06 | 28.6% (2/7) | L-glutamine, phosphate |
| L-glutamine Biosynthesis II (tRNA-dependent) | 7.90E-06 | 18.2% (2/11) | L-glutamine, phosphate |
| Sphingosine and Sphingosine-1-phosphate Metabolism | 2.20E-05 | 11.1% (2/18) | phosphate, O-phosphorylethanolamine |
| 5-aminoimidazole Ribonucleotide Biosynthesis I | 2.20E-05 | 11.1% (2/18) | L-glutamine, phosphate |
| Uridine-5'-phosphate Biosynthesis | 2.45E-05 | 10.5% (2/19) | L-glutamine, phosphate |
| **From the CRS with SD overlap** | | | |
| Phosphatidylethanolamine Biosynthesis III | 1.44E-06 | 40.0% (2/5) | ethanolamine, L-serine |
| Serine Biosynthesis | 1.31E-05 | 14.3% (2/14) | L-serine, phosphate |
| Selenocysteine Biosynthesis II (Archaea and Eukaryotes) | 1.72E-05 | 12.5% (2/16) | L-serine, phosphate |
| Sphingosine and Sphingosine-1-phosphate Metabolism | 2.20E-05 | 11.1% (2/18) | phosphate, O-phosphorylethanolamine |
| Folate Polyglutamylation | 2.20E-05 | 11.1% (2/18) | L-serine, phosphate |
| **From the 4 rat model overlap** | | | |
| Sphingosine and Sphingosine-1-phosphate Metabolism | 6.11E-06 | 11.1% (2/18) | phosphate, O-phosphorylethanolamine |
| γ-glutamyl Cycle | 1.62E-05 | 6.9% (2/29) | phosphate, oxoproline |
| S-methyl-5’-thioadenosine Degradation II | 1.34E-03 | 16.7% (1/6) | phosphate |
| Glutamine Biosynthesis I | 1.56E-03 | 14.3% (1/7) | phosphate |
| NADH Repair | 1.79E-03 | 12.5% (1/8) | phosphate |

Due to the technical properties of mass spectrometry, we did not separate two isomers of amino acids (i.e., L-glutamine or D-glutamine). However, Ingenuity Pathway Analysis software automatically identified the amino acids as L-isomers. CUMS, chronic unpredictable mild stress; LH, learned helplessness; CRS, chronic restraint stress; SD, social defeat.
